# Formulation and Characterization of Benzoyl Peroxide Gellified Emulsions

**DOI:** 10.3797/scipharm.1206-09

**Published:** 2012-08-31

**Authors:** Naresh Kumar Thakur, Pratibha Bharti, Sheefali Mahant, Rekha Rao

**Affiliations:** 1 MM College of Pharmacy, MM University, Mullana (Ambala) – 133207, India.; 2 Department of Biotechnology, Panjab University, Chandigarh – 160014, India.; 3 Department of Pharmacy, Government Polytechnic, Uttawar (Faridabad) – 121103, India.

**Keywords:** Emulgel, Acne vulgaris, Topical drug delivery, Histopathological study

## Abstract

The present investigation was carried out with the objective of formulating a gellified emulsion of benzoyl peroxide, an anti-acne agent. The formulations were prepared using four different vegetable oils, viz. almond oil, jojoba oil, sesame oil, and wheat germ oil, owing to their emollient properties. The idea was to overcome the skin irritation and dryness caused by benzoyl peroxide, making the formulation more tolerable. The gellified emulsions were characterized for their homogeneity, rheology, spreadability, drug content, and stability. *In vitro* permeation studies were performed to check the drug permeation through rat skin. The formulations were evaluated for their antimicrobial activity, as well as their acute skin irritation potential. The results were compared with those obtained for the marketed formulation. Later, the histopathological examination of the skin treated with various formulations was carried out. Formulation F3 was found to have caused a very mild dysplastic change to the epidermis. On the other hand, the marketed formulation led to the greatest dysplastic change. Hence, it was concluded that formulation F3, containing sesame oil (6%w/w), was the optimized formulation. It exhibited the maximum drug release and anti-microbial activity, in addition to the least skin irritation potential.

## Introduction

Acne vulgaris is presumably the most common skin ailment, characterized by inflammation of the pilosebaceous glands. Owing to its wide prevalence, it is often referred to as a physiological condition. Acne vulgaris is known to affect 85% of the young population between 12 to 24 years of age. In addition to this, 8% of the adults in the age group of 25 to 34 years and 3% of adults aged 35 to 44 years also suffer from acne [1]. The disease may vary in its intensity from a papule to cystonodular acne. The management of the disease takes into account the severity of the disease, as well as patient factors like age, skin type, lifestyle, menstrual regularity, and so on [2].

Pharmacotherapy of acne includes a number of drugs administered orally or topically. Topical administration of anti-acne agents comprises an important part of the therapy. Topical delivery is not only devoid of systemic toxicity caused by the drug, but also makes the drug available directly at the site of application [3].

Benzoyl peroxide (BPO) is one of the most widely prescribed drugs in acne therapy. It is an anti-bacterial agent which releases free radical oxygen species capable of oxidizing bacterial proteins. Additionally, it also has a mild keratolytic effect [4]. It is commonly available as cream and gel, either alone or in combination with other drugs. However, the drug is known to cause considerable skin dryness and irritation, sometimes leading to the discontinuation of the treatment [5, 6]. In 2010, Steve Feldman and Diana Chen conducted an internet-based survey to study the impact of dryness and irritation from acne treatment on patient practices. A total of 200 subjects, using a commercially available Clindamycin-BPO (5%) fixed combination product, participated in the survey. Around 55% of the subjects were reported to have been extremely bothered by having dry and flaky skin, 44% were reported to have irritated skin, and 39% and 37% reported itching and redness, respectively. Furthermore, it was reported that 33% of the subjects did not use the product optimally, 32% used it less than recommended, 32% stopped using the product from time to time, 10% of the patients stopped using it altogether, and 41% applied moisturizers to counteract the dryness and redness [7].

The present study was attempted with the objective to develop a topical formulation of BPO in the form of a gellified emulsion. A review of the past research work on BPO formulations affirmed that a gellified emulsion has not yet been prepared with this drug. Although, many scientists and physicians have expressed concern about the skin irritation caused by this drug, the problem has not been completely addressed. A gellified emulsion of BPO with emollient vegetable oils represents a cheaper and better alternative to combat skin dryness and irritation, as opposed to other formulations like BPO foams. Gellified emulsions combine the properties of both, emulsion and gel, thereby exhibiting improved stability and elegance. Moreover, such formulations also alter drug release characteristics, aside from being greaseless, thixotropic, easily spreadable, easily removable, non-staining, bio-friendly, emollient, and transparent. Hydrophobic drugs like benzoyl peroxide are particularly suited for such a formulation as the oil-in-water type gellified emulsion [8].

The emulsifying agent, the oil phase, and a gelling agent constitute the major components of a gellified emulsion formulation, as they contribute to the physicochemical properties of the final preparation. For the purpose of this study, various vegetable oils, namely, almond oil, jojoba oil, sesame oil, and wheat germ oil were used as the oil phase. These oils were selected owing to their characteristic properties. The following points account for the selection of the oil phase:

○ The main constituents of almond oil are glycerides of oleic and linoleic acid, phytosterols, α-tocopherol, and vitamin K. Fatty acids are metabolized in the skin when applied topically, thereby normalizing the cell lipid layer and improving the water retention capability. α-Tocopherol improves water-binding ability, improving the appearance of rough and damaged skin. In addition to these, it does not clog pores or leave the skin oily [9].
○ Wheat germ oil is one of the richest sources of Vitamin A, E, and D. It has a high content of lecithin and proteins. It is extensively used for external application as it helps in combating skin irritation, including skin dryness and cracking. It is also a good source of fatty acids and minimizes symptoms of dermatitis [10].
○ Jojoba oil is a wax ester consisting of a complex mixture of naturally-occurring long-chained linear esters. It also contains tocopherols, sterols, and other unsaponifiable matter. It is an excellent moisturizer that does not block skin pores and helps restore normal oil balance of the skin. The oil is stable to oxidation and imparts photo-protection. It also possesses anti-inflammatory and antibacterial properties, thereby, making it useful in acne treatment [11, 12].
○ Sesame oil is mostly composed of triglycerides of singly unsaturated oleic acid and the doubly unsaturated linoleic acid, besides a small percentage of saturated fats. It contains powerful antioxidants. The most important ingredient that makes it very beneficial for skin is vitamin E, which imparts exceptional moisturizing and emollient properties. Moreover, it is valuable in the prevention and cure of acne due to its oil-pulling property [13, 14].
○ The idea behind the incorporation of these vegetable oils was to counteract the skin dryness and irritation caused by the conventional BPO formulations, making the treatment more tolerable for the patient.
○ Span 60 and Tween 20 were added as emulsifying agents. They belong to the category of non-ionic surfactants. They are non-toxic and have been successfully used in the formulation of gellified emulsions. Carbopol 940, a cross-linked polyacrylate polymer, was added as a gelling agent. It is a rheology modifier and is extensively used in the formulation of hydroalcoholic gels and creams. Butylated hydroxyl toluene and disodium EDTA were incorporated as an antioxidant and a chelating agent, respectively. Methyl paraben and propyl paraben were included as preservatives, as certain components of the formulations are prone to microbial attack.

Finally, the prepared formulations were subjected to various evaluation tests to assess their properties such as viscosity, pH, spreadability, drug content, stability, *in vitro* drug release, and acute skin irritation potential. The prepared formulations were compared to a commercial formulation i.e. 5% BPO gel, with regard to its antimicrobial activity against the acne-causing bacteria *Staphylococcus aureus.*

## Experimental

The following materials were used in this study: Benzoyl peroxide (Oxford Laboratory, New Delhi), Carbopol 940, Tween 20, Span 60, Propylene glycol, and Methyl paraben (Qualikames Fine Chem. Pvt. Ltd., New Delhi), Propyl paraben and Butylated Hydroxy Toluene (CDH Laboratory Reagent, New Delhi). Almond oil, Jojoba oil, Sesame oil, and Wheat germ oil were received as gift samples from Katyani Exports, New Delhi.

### Drug-excipient compatibility study

Infrared spectroscopy of the drug and excipients was carried out to check for any drug-excipient interactions. The IR spectral analysis of benzoyl peroxide was carried out with the Fourier Transform Infrared spectrophotometer using a KBr pellet, in the scanning range of 2000 to 4000 nm.

### Preparation of gellified emulsions [15–18]

The steps involved in the preparation of the gellified emulsion include the preparation of the emulsion phase, followed by the addition of the emulsion into an aqueous solution of the gelling agent, to form a semisolid formulation. The oil phase of the emulsion was prepared by dissolving the lipophilic surfactant (Span 60) in vegetable oil while the hydrophilic surfactant (Tween 20) was dissolved in distilled water to obtain the aqueous phase. The drug was dissolved in propylene glycol, and then methyl paraben, propyl paraben, and disodium EDTA were added to the solution. Butylated hydroxy toluene was added to the oil phase. Both the oil and aqueous phase were heated to 60–65°C, followed by the addition of the oil phase into the aqueous phase with constant stirring, until it cooled to room temperature. The obtained emulsion was mixed with the weighed quantity of the gelling agent, in the ratio 1:1, to obtain an elegant gellified emulsion.

### Composition of gellified emulsions

The compositions of various batches are given in table 1:

### Evaluation of benzoyl peroxide gellified emulsion formulations

Evaluation of gellified emulsions included the following tests:

#### Homogeneity study [19]

The prepared gellified emulsions were inspected visually for their color and homogeneity.

#### pH determination [20]

The pH of the prepared gellified emulsions was determined by using a digital pH meter. 1g of the gellified emulsion was stirred in distilled water, until a uniform dispersion was formed. It was kept aside for 2 hours. The volume was then made up to 100 ml, i.e. 1% solution of prepared formulation. Then, pH measurement was performed. The test was performed in triplicate using a digital pH meter and the mean was calculated.

#### Viscosity measurements of gellified emulsions [21, 22]

The gellified emulsion formulations were assessed for their viscosity using a Brookfield viscometer (Brookfield LV, spindle no. 63), at 25°C at an rpm of 50.

#### Drug content determination [23, 24]

The gellified emulsion formulations were dissolved in ethanol and the volume was made up to 100 ml with ethanol. The resultant solution was suitably diluted with ethanol and the absorbance was measured at 234 nm, using the Shimadzu-1800 UV-Visible spectrophotometer. The drug content was determined from the calibration curve of benzoyl peroxide. This test was performed in triplicate and the average drug content for each formulation was calculated.

#### In vitro skin permeation study [24–28]

An essential parameter in the evaluation of the drug delivery is the rate at which the drug is released from the carrier. Skin permeation studies with benzoyl peroxide gellified emulsion formulations were carried out. Before carrying out the study, approval was obtained from the Institutional Animal Ethical Committee, MM University, Mullana (MMCP/IEC/11/09).

The modified Franz diffusion cell was used to carry out the skin permeation studies. The full thickness of the abdominal skin of male albino rats, weighing 140 to 200 g, was used for the skin permeation and deposition studies. Briefly, to obtain skin, animals were sacrificed. Hairs from the abdominal region were carefully removed and an excision of the skin was made. The dermal side of the skin was thoroughly cleaned of any adhering tissues. The dermis part of the skin was wiped three or four times with a wet cotton swab, soaked in isopropanol, to remove any adhering fat material. The skin specimens were cut into the appropriate size, after carefully removing subcutaneous fat and washing with normal saline. The skin was mounted in a modified Franz diffusion cell, maintained at 37°C. A known quantity of the formulation was spread uniformly on the skin, on the donor side. Phosphate buffer saline solution (pH 7.4) was used as the receptor medium. At predetermined time intervals (1, 2, 3, 4, 5, 6 hours), 3 ml samples of the receptor medium were withdrawn and suitably diluted. They were filtered for analysis and replaced with an equal volume of the buffer solution to maintain a constant volume. The absorbance of the collected samples was measured by the UV spectrophotometer at λ_max_ of 234 nm, using the same buffer solution as a control medium. The *in vitro* skin permeation studies were carried out in triplicate for each formulation. Thereafter, the mean values and standard deviations for the amount of drug permeated were calculated and used for further calculations.

#### Spreadability study [29–32]

The spreadability of the formulations was determined by using an apparatus suggested by Mutimer et al. (1956), which was suitably modified in the laboratory and used for the study. By this method, the spreadability was measured on the basis of ‘slip and drag’ characteristics of the formulation. A ground glass slide, measuring 20 cm in length, was fixed on the table. An excess of gellified emulsion (about 2 g) was placed on this ground slide. The gellified emulsion was then sandwiched between this slide and another glass slide, having the dimensions of a fixed ground slide and provided with the hook. A 500 gm weight was placed on the top of the two slides for 5 minutes, to expel air and to provide a uniform film of the gellified emulsion between the slides. Excess of the gellified emulsion was scraped off from the slides’ edges. The top plate was then subjected to a pull with 50 g of weight tied on the upper slide, at a distance of 7 cm. Lesser the time taken by the slides to move the specified distance of 7 cm, the better the spreadability of the gellified emulsion.

The spreadability of the prepared gellified emulsion was calculated by using the following formula:

S=M×L/T

where S is the spreadability (g.cm/sec), M represents the weight tied to the upper slide (g), L represents the length moved by the glass slide (cm), and T stands for the time taken to separate the slides completely from each other (sec).

#### Stability studies [33, 34]

All of the formulations were subjected to stability testing at different temperature conditions (4°C and room temperature, 25°C) for 3 months. Parameters such as pH, viscosity, spreadability, drug content, consistency, and phase separation were examined at fortnightly intervals.

#### Antimicrobial testing using disc diffusion method [35–37]

All of the prepared formulations were checked for their antimicrobial activity against *Staphylococcus aureus,* one of the bacteria responsible for causing acne, using the disc diffusion method.

The Kirby-Bauer test, also known as the disk-diffusion method, is the most widely used method to determine the sensitivity or resistance of pathogenic aerobic and facultative anaerobic bacteria to various antimicrobial compounds. This method relies on the inhibition of bacterial growth measured under standard conditions. For the purpose of this test, a culture medium, specifically the Mueller-Hinton agar medium, is uniformly and aseptically inoculated with the test organism. Then, filter paper discs impregnated with a specific concentration of a particular antibiotic, are placed on the medium. The organism grows on the agar plate while the antibiotic “works” to inhibit the growth. If the organism is susceptible to a specific antibiotic, there will be no growth around the disc containing the antibiotic. Thus, a “zone of inhibition” can be observed and measured to determine the susceptibility to an antibiotic for that particular organism.

To check the antimicrobial activity of the prepared formulations and to compare it with that of the marketed formulation, a concentration of 10 mg/ml of all of the prepared formulations and the marketed formulation was prepared. The concentrations of each formulation were incorporated into the filter paper discs. Later, the disc of each formulation was placed into the agar medium for 24 hours at 37°C. It gave a clear zone of inhibition around the disc, indicating the antimicrobial activity of the formulations. The zones of inhibition so obtained were measured and the results were compared. This study was performed in triplicate for each formulation.

#### Acute skin irritation study [35–39]

The primary skin irritation test was performed on male albino rats, weighing about 150–200 g. A set of six rats was used in the study for each formulation. The animals were maintained on standard animal feed and free access to water. The animals were kept under standard laboratory conditions. The dorsal hairs on the back of the rats were clipped off one day prior to the study. 50 mg of the different formulations was applied over a 1 cm^2^ area of intact skin on different animals. After the gellified emulsion was applied to the skin of rabbits, the animals were returned to the cages. After 48 hours of exposure, the gellified emulsion was removed. The test site was wiped with tap water to remove any remaining residue. Undesirable skin changes, i.e., change in color and change in skin morphology were checked.

#### Histopathological examination of the skin specimens [40]

Formulation F1 and F3 were selected for the histopathological study on the basis of the results obtained from the skin irritation study. The study was carried out on healthy male albino rats (150–200 g). The dorsal skin area of 2cm^2^ was shaved one day prior to the study. The prepared formulations, F1 and F3 and the marketed formulation were applied to the shaven skin. The rats were sacrificed and the skin samples were excised from the treated and untreated areas (control). Each specimen was stored in 10% formalin solution. The specimens were cut into vertical sections, stained with hematoxylin-eosin and examined under the Nikon Pentahead Microscope. The untreated intact skin served as a control for comparison.

#### Statistical Analysis

Statistical analysis was performed using Student’s paired t-test using Graphpad Version 2.01, San Diego, CA. The data was considered significant at (P< 0.05).

## Results and Discussion

BPO emulgels were successfully formulated using different vegetable oils as the oil phase. The resulting formulations were found to fulfill all criteria for a satisfactory topical formulation, as is evident from the following discussion.

### Homogeneity study

The prepared gellified emulsion formulations were white, viscous, and creamy preparations, with a smooth and homogeneous appearance, as given in table 2.

Incorporation of propylene glycol as a humectant not only provided the optimum spreadability to the product, but also improved the aesthetic appearance. Carbopol 940 as a gelling agent helped to achieve the desired viscosity, thereby affecting the homogeneity as well as the spreadability of the final preparations. There was no sign of phase separation in any of the preparations.

### pH determination

The pH of all of the gellified emulsions was found to be in the range of 6.2 to 6.5, which lies in the normal pH range of the skin. Triethanolamine was added to the formulations in order to obtain pH in the desired range.

### Viscosity of gellified emulsions

All of the prepared formulations possessed optimum viscosity. Since, the type and quantity of the gelling agent in each formulation was the same, inclusion of a different vegetable oil seems to have brought about some difference in the viscosity of the gellified emulsions. While F2 was the most viscous formulation, F3 had the least viscosity.

### Percent drug content determination

The percent drug content in gellified emulsions was found to fall in the range of 104 to 100.

### Spreadability study

The values of the spreadability indicated that the gellified emulsions were easily spreadable by small amount of shear. Formulation F1 gave the highest value for spreadability.

### In vitro permeation study

In order for a topical formulation to be effective in treating a cutaneous disease, an optimum drug release is the prerequisite. Hence, it is imperative to determine the rate and extent of drug release from the gellified emulsion base. Various components of the gellified emulsion are known to have an impact on the drug release. Mohamed MI, from his research on chlorphensin emulgel, reported that the drug release is affected by the concentration of the emulsifying agent, concentration of the oil phase, and the type of gelling agent [15]. Since, the present study was performed with a single gelling agent and the same combination of emulsifying agents, incorporation of a different oil in each formulation would have affected the drug release characteristics of the final preparations.

The following observations were recorded:

The *in vitro* release of the drug from the formulation F3 was found to be better as compared to the other gellified emulsions. The reason for good *in vitro* release may be the low viscosity of the sesame oil formulation.
The drug release from formulation F4, i.e. jojoba oil, also showed good *in vitro* release, which may be attributed to the low viscosity of F4.
The drug release from the different formulations followed the order: F3>F4>F1>F2.

These observations are supported by the conclusions drawn from the research carried out in the past. Mura et al. reported that the drug release from different hydrophilic ointment bases was inversely related to their viscosity. When drug diffusion is the rate-limiting step, the viscosity of the matrix is a significant factor that controls the rate of drug release [16].

**Fig. 1.**
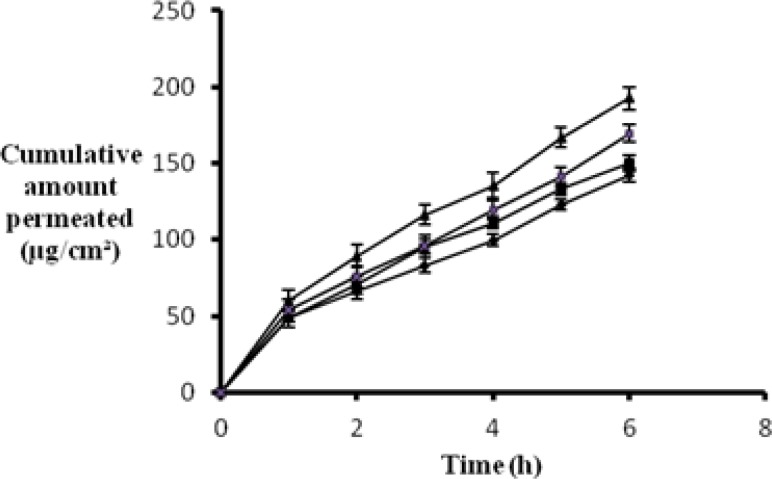
Release profile of different BPO gellified emulsions

Figure 1 shows a comparative plot of the *in vitro* release profile of the different gellified emulsions.

### Stability study

All of the prepared gellified emulsions were found to be stable upon storage for three months. No change was observed in their physical appearance, homogeneity, pH, and drug content. The stability study data of different gellified emulsions is tabulated as follows:

### Antimicrobial testing using the disc diffusion method

The results obtained from the antimicrobial study showed that all developed formulations have an inhibitory effect on the *Staphylococcus aureus.* The antimicrobial activity of the all of the formulations was compared with that of the marketed formulation (5% benzoyl peroxide gel) and the control. The measured zones of inhibition followed the order: F3 > F4 > F5 > F2 > F1 > F6. (F5 and F6 stand for the marketed formulation and the control, respectively).

### Acute skin irritation study

The skin irritation study was performed on male albino rats. This test is significant as it gives an idea about the emollient properties of the prepared formulations against the skin irritation caused by BPO. The results of skin irritation were assessed by visual inspection. They are given in table 5:

As is evident from the above findings, the formulation F3 caused no skin irritation. Formulations F1, F3, and F5 were further subjected to the histopathological study, with the objective to confirm the results of the skin irritation study.

### Histopathological examination of skin specimens

**Fig. 4.**
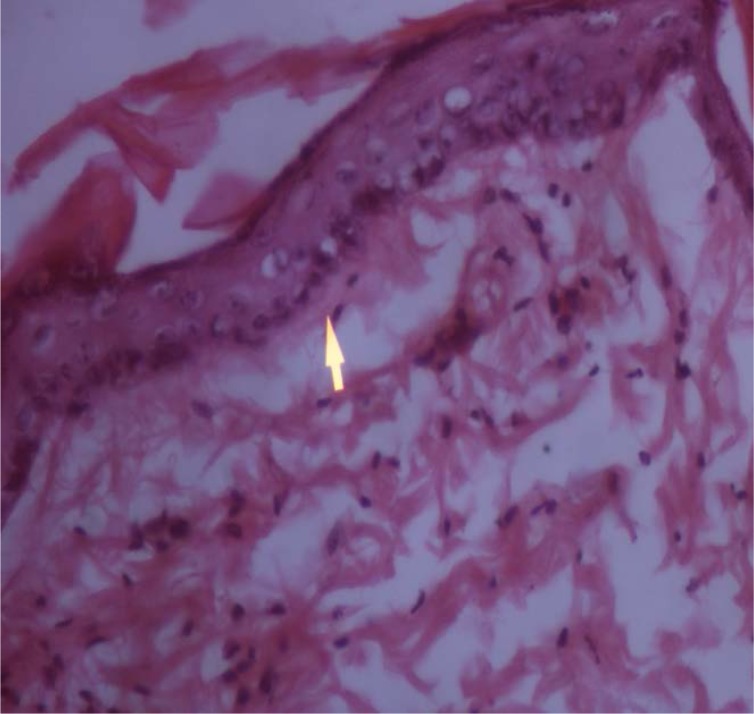
Skin treated with F1

**Fig. 5.**
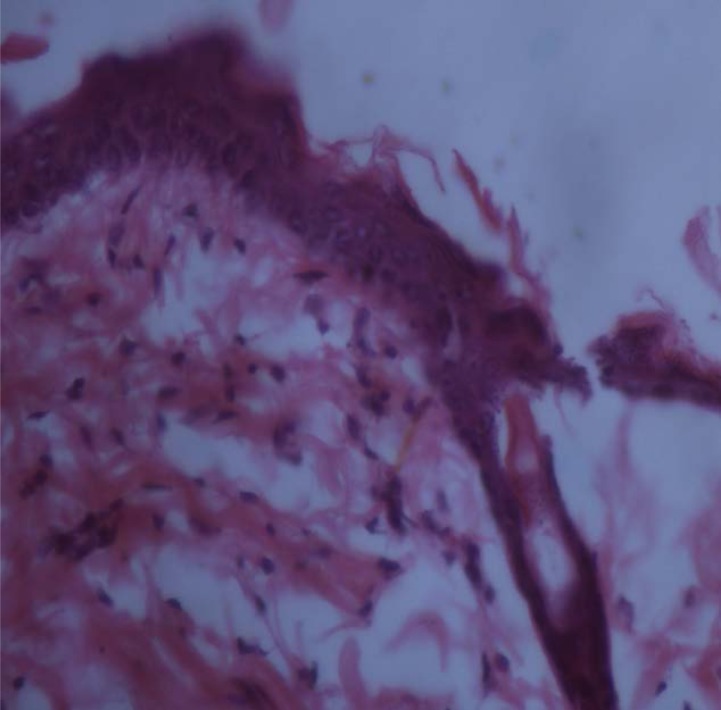
Skin treated with F3

**Fig. 6.**
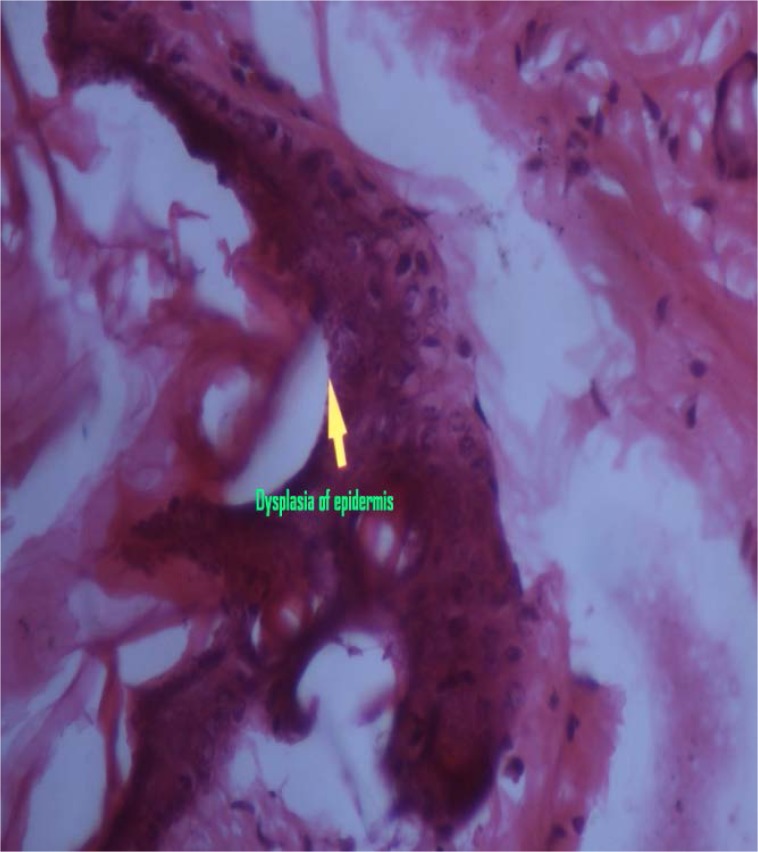
Skin treated with F5

**Fig. 7.**
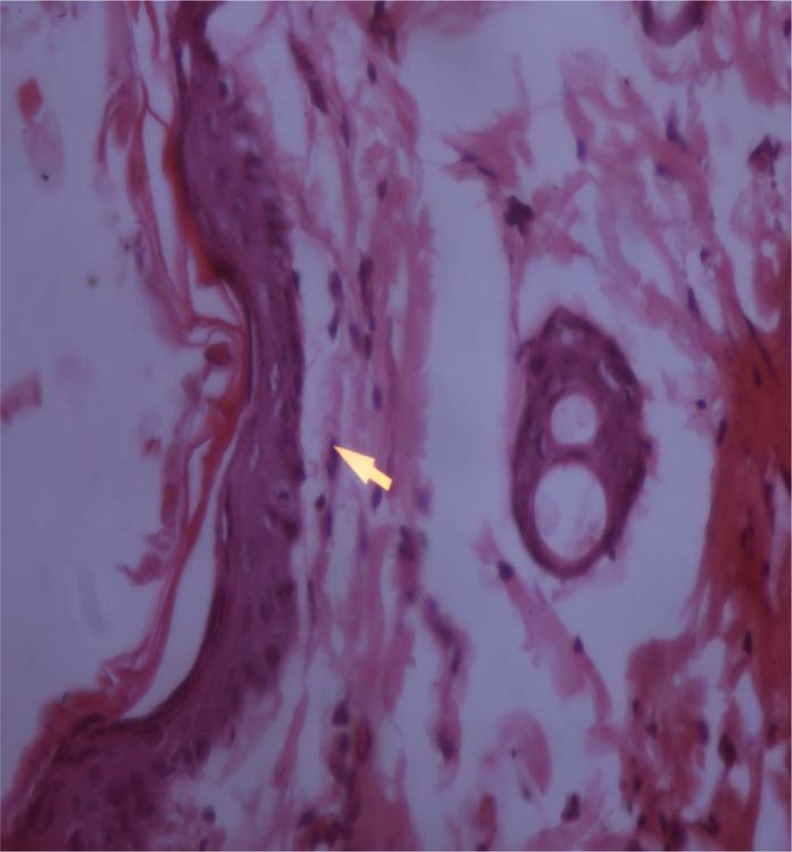
Untreated skin (Control)

Histopathological examination of the skin specimens treated with gellified emulsions revealed the following:

Dysplastic changes were observed in the skin treated with formulation F1 (almond oil). While formulation F3 (sesame oil)-treated skin showed only mild thickening of the epidermis, skin treated with the marketed formulation (F5) showed dysplastic changes as well as a basal vacuolar change in the epidermal layer. These were compared with the untreated skin which was kept as the control. The photomicrographs of the various skin specimens are given in the figures 4–7. Hence, from the histopathological examination, it is clear that formulation F3 caused the least irritation to the skin. Furthermore, all of the formulations were found to be better than the marketed formulation. This supports the observations recorded in the skin irritation study.

## Conclusion

The coming years will witness an extensive use of topical drug delivery systems as these ensure better patient compliance and constitute an effective treatment option, devoid of systemic toxicity. Gellified emulsions are relatively newer and better topical drug delivery systems since they enjoy the advantages of both emulsions and gels. They enable the poorly aqueous-soluble drugs to be loaded into a hydrophilic gel base.

Benzoyl peroxide was successfully incorporated into the gellified emulsion bases consisting of different vegetable oils. At the end of the investigation, it was concluded that the incorporation of sesame oil in the concentration of 6% w/w in the formulation resulted in an optimized gellified emulsion of benzoyl peroxide. The formulation not only exhibited satisfactory physical properties and stability, but also showed maximum drug release. The formulation was also found to possess the greatest anti-microbial activity against S. aureus. It caused very little irritation to the skin, as revealed from the acute skin irritation study and the histopathological examination of the rat skin. In this aspect, it was found to be far superior to the marketed formulation, thus, fulfilling the objective of the study.

## Figures and Tables

**Fig. 2.**
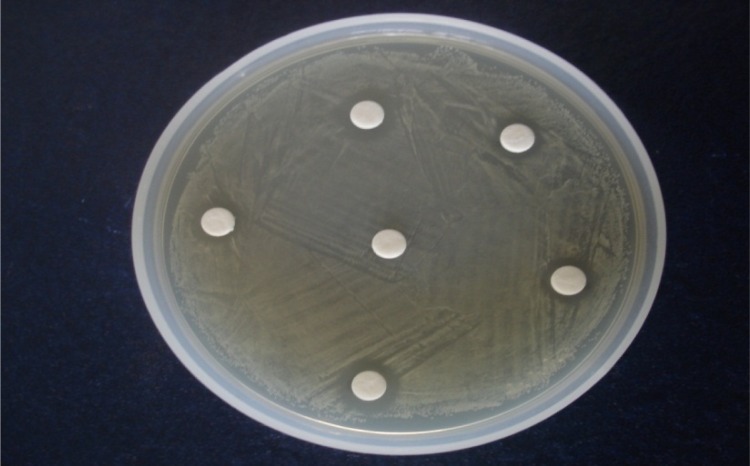
Antimicrobial activity of BPO in gellified emulsions against *S. aureus* (showing zones of inhibition)

**Fig. 3.**
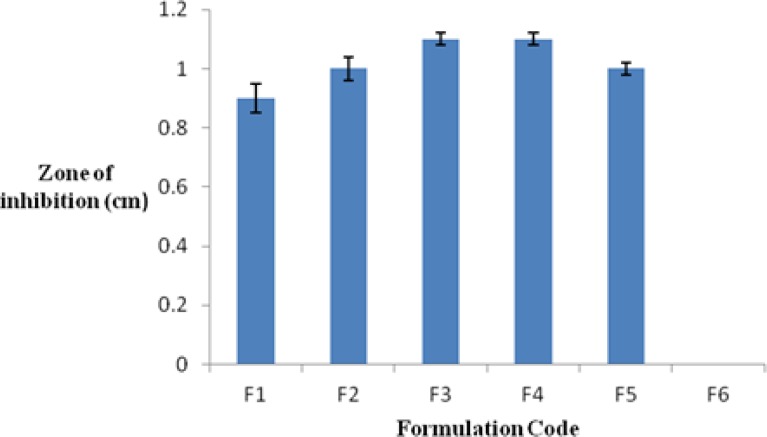
Zones of inhibition obtained for different BPO gellified emulsions

**Tab. 1.** Composition and codes of BPO gellified emulsions

**S. No.**	**Ingredients**	**Quantity (g)**			
		**F1**	**F2**	**F3**	**F4**
1	Carbopol 940	1.5	1.5	1.5	1.5
2	Benzoyl peroxide	5	5	5	5
3	Almond oil	6	–	–	–
4	Wheat germ oil	–	6	–	–
5	Sesame oil	–	–	6	–
6	Jojoba oil	–	–	–	6
7	Tween 20	2	2	2	2
8	Span 60	2.5	2.5	2.5	2.5
9	Propylene glycol	5	5	5	5
10	Methyl paraben	0.03	0.03	0.03	0.03
11	Propyl paraben	0.01	0.01	0.01	0.01
12	Purified water	Upto 100	Upto100	Upto100	Upto100
13	Disodium EDTA	0.1	0.1	0.1	0.1
14	Butylated hydroxyl toluene	0.1	0.1	0.1	0.1

**Tab. 2.** Homogeneity of BPO gellified emulsions

**Formulation**	**Color**	**Homogeneity**	**Texture**
F1	White	Homogeneous	Smooth
F2	White	Homogeneous	Smooth
F3	White	Homogeneous	Smooth
F4	White	Homogeneous	Smooth

**Tab. 3.** Observations for different physical parameters of BPO gellified emulsions

**Formulation**	**pH**	**Viscosity (mPas)**	**Percent drug content**	**Spreadability (g.cm /sec.)**
F1	6.31±0.012	11147±20.95	104±2.102	13.47±0.66
F2	6.56±0.025	11202±21.65	100±1.572	13.32±0.75
F3	6.25±0.026	10390±28.25	102±0.725	10.74±0.51
F4	6.29±0.014	10588±25.25	103±1.045	11.95±0.61

**Tab. 4.** Stability study data of different BPO gellified emulsions

**Formulation**	**Day**	**Color**		**Homogeneity**		**pH**		**% Drug content**	

		**4°C**	**25°C**	**4°C**	**25°C**	**4°C**	**25°C**	**4°C**	**25°C**
F1	0	White	White	+++	+++	6.31	6.31	104	104
	30	White	White	+++	+++	6.26	6.26	104	103
	60	White	White	+++	+++	6.22	6.21	102	101
	90	White	White	+++	+++	6.2	6.18	98	99

F2	0	White	White	+++	+++	6.56	6.56	100	100
	30	White	White	+++	+++	6.5	6.5	100	99
	60	White	White	+++	+++	6.4	6.38	98	99
	90	White	White	+++	+++	6.36	6.37	96	97

F3	0	White	White	+++	+++	6.25	6.25	102	102
	30	White	White	+++	+++	6.23	6.18	102	101
	60	White	White	+++	+++	6.19	6.2	99	97
	90	White	White	+++	+++	6.18	6.18	96	96

F4	0	White	White	+++	+++	6.19	6.19	103	103
	30	White	White	+++	+++	6.14	6.16	102	103
	60	White	White	+++	+++	6.12	6.12	98	101
	90	White	White	+++	+++	6.09	6.12	96	98

**Tab. 5.** Observations from skin irritation study

**S. No.**	**Formulation**	**Observation**
1	F1	Redness or dryness observed
2	F2	Redness observed
3	F3	No sign of redness or dryness observed
4	F4	Little sign of redness observed
5	F5	Redness and dryness observed
